# State-of-the-Art Research: Current Developments in CT Imaging

**DOI:** 10.3390/diagnostics13132305

**Published:** 2023-07-07

**Authors:** Christian Booz

**Affiliations:** Department of Diagnostic and Interventional Radiology, University Hospital Frankfurt, 60590 Frankfurt, Germany; christian.booz@kgu.de

This Special Issue of *Diagnostics* entitled “Advances in CT Images” provides an interesting selection of articles on recent technical developments in CT imaging with a special focus on spectral imaging, including dual-energy CT (DECT) and photon-counting CT (PCCT). 

In recent years, the use of spectral CT algorithms, such as material decomposition and virtual monoenergetic imaging (VMI), has expanded the capabilities of CT imaging, opening up new possibilities for enhanced diagnostic accuracy and a more comprehensive understanding of tissue characteristics, thus improving patient safety through reduced contrast medium doses. This Special Issue particularly focuses on selecting manuscripts that clearly highlight these advantages. Bucolo et al. [1] conducted a study on VMI in the lower extremity assessment of patients with diabetes mellitus. The authors showed the benefit of using 40 and 55 keV-VMI+ images, which provided the highest objective and subjective image quality parameters, with the potential to reduce contrast medium doses for diabetic patients suffering from renal impairment. Dillinger et al. [2] evaluated the impact of the novel PCCT VMI algorithm in the assessment of abdominal vessels, showing the best objective and subjective image quality at 60–70 keV concerning vessel contrast irrespective of vessel size. Moreover, VMI could improve the assessment of venous structures, as shown by Martin et al. [3]. Their study demonstrated that 40-keV VMI+ DECT showed the best contrast-to-noise ratio, image quality, and diagnostic accuracy of portal venous thrombosis compared to other DECT reconstructions. 

Another highly interesting application of VMI was evaluated by Estler et al. [4] in the detection of hepatocellular carcinoma (HCC) using PCCT. Their findings showed that the arterial phase, particularly at 40-keV, provides a higher lesion-to-background ratio of HCC lesions, although no significant difference was observed in subjective readings. Furthermore, low-keV VMI DECT proved to be an effective method for observing the pancreatic duct system. Zhang et al. [5] demonstrated the association between pancreatic duct variation and acute/chronic pancreatitis as well as duodenal papillary carcinoma, which was better assessed by using low-keV VMI+ reconstructions. 

The DECT material decomposition algorithm can be used to assess iodine uptake and fat fraction in pathological tissue. Mahmoudi et al. [6] investigated these parameters in patients with acute pancreatitis, aiming to explore the correlation and diagnostic accuracy of DECT-derived imaging biomarkers for differentiating various levels of severity. Their finding suggested that DECT iodine quantification could be a useful tool for predicting the severity and prognosis of acute pancreatitis. 

Winkelmann et al. [7] used DECT applications to discriminate between intraperitoneal hematomas and physiological bowel structures. In particular, virtual non-contrast imaging and iodine maps showed an improvement in differentiation between intraperitoneal hematomas and adjacent bowel structures when compared to single-energy CT.

Moreover, DECT-based material decomposition enabled differentiation of the composition of urine sedimentation and stones. As proven by Booz et al. [8], DECT imaging can be used in the differentiation of uric acid from calcium, providing valuable information for sedimentation/stone composition analysis and aiding in treatment decisions. 

A novel DECT post-processing algorithm exists which enables color-coded mapping of collagenous structures, facilitating the assessment of ligament structures. Gruenewald et al. [9] used this algorithm to evaluate the integrity of distal tibiofibular syndesmosis in patients with acute trauma, with the authors finding substantially higher diagnostic accuracy and confidence compared to conventional greyscale CT.

In the meantime, technological developments have led to the development of ultra-high-resolution CT (UHR-CT) scanners with a slice thickness of up to 0.25 mm. Altmann et al. [10] showed that UHR-CT with a deep-learning-based image reconstruction engine provides superior image quality at a markedly lower radiation dose compared to normal-resolution CT in head and neck assessment, suggesting an improvement in the detection of pathologies in this field. Moreover, Jabas et al. [11] evaluated the efficacy of a single-energy metal artifact reduction algorithm in UHR-CT angiography in patients with intracranial coils and clips, with the results demonstrating a significant reduction in metal artifacts with a consequent improvement in image quality and diagnostic confidence. 

In present times, the role of radiological imaging is increasingly important in emergencies, even in pediatric patients. Lanzafame et al. [12] described two cases of button battery ingestion in healthy infants, highlighting how, in one of the two cases, CT imaging successfully detected an aorto-oesophageal fistula, a complication derived from the corrosive effect of the disc battery, where the angiographic method had failed.

I have great confidence in the potential of this Special Issue as an impactful scientific tool to showcase the latest advancements in CT imaging technology. It will serve as a comprehensive resource for radiologists, researchers, and clinicians, highlighting the main potentials of spectral imaging and its significant clinical implications, overcoming the limitations of conventional CT. Moreover, it will provide future perspectives through the application of new protocols that may reduce contrast medium and radiation doses. Furthermore, its ability to analyze tissue composition solidifies its role in shaping treatment decisions and driving advancements in the field.
